# Differences in Anxiety and Depression Among Migrant and Non-Migrant Primary School Children in The Netherlands

**DOI:** 10.1007/s10578-022-01454-0

**Published:** 2022-11-02

**Authors:** Mia P Kösters, Mai JM Chinapaw, Marieke Zwaanswijk, Marcel F van der Wal, Hans M. Koot

**Affiliations:** 1 Healthy Living Department, Public Health Service of Amsterdam, Amsterdam, The Netherlands; 2 Amsterdam UMC location Vrije Universiteit Amsterdam, dept Public and Occupational Health, Amsterdam, The Netherlands; 3 Amsterdam Public Health Research Institute, Amsterdam, The Netherlands; 4 Dutch Knowledge Centre for Child and Adolescent Psychiatry, Utrecht, The Netherlands; 5 Department of Clinical, Neuro‑ and Developmental Psychology, Amsterdam Public Health Research Institute, Amsterdam, The Netherlands; 6 Department of Clinical, Neuro‑ and Developmental Psychology, Vrije Universiteit Amsterdam, Van der Boechorststraat 7, 1081 BT Amsterdam, The Netherlands

**Keywords:** Anxiety, Depression, Child, Migrant background

## Abstract

This is the first Dutch study investigating symptoms of five DSM-IV-classified anxiety disorders and depression in a large sample of pre-adolescent children with and without a migration background, adjusting for socioeconomic position (SEP) and social preference. Both are potential explanatory factors for differences in mental health among migrant children. We measured anxiety and depression scores with the self-report Revised Child Anxiety and Depression Scale (RCADS) in 2063 children (aged 8–13 years, 55% girls) in the Netherlands. Surinamese/Antillean, Turkish, and Moroccan children reported significantly higher anxiety scores than Dutch children. SEP and peer rejection partly explained higher anxiety scores. Surinamese/Antillean and Turkish children reported comparable depression scores to Dutch children, but Moroccan children reported lower depression scores after adjusting for SEP and peer rejection. Girls reported higher anxiety and depression levels across all four subgroups. Although differences between children with or without a migration background were small, these may increase in later life as the prevalence of anxiety and depression increases with age.

## Introduction

In European countries, the population of migrant youth – first generation foreign-born or second generation native-born with foreign-born parents – is expanding [1]. Various European cities have become or are close to becoming majority-minority cities, i.e. cities in which the majority of the population consists of people from ethnic minorities [2]. This is also the case for Amsterdam, the Netherlands. In Amsterdam elementary schools, the share of children from migrant groups (henceforward migrant children) has been about 60% of the total elementary school population over the last decade [3, 4].

In general, the literature states that belonging to a migrant minority increases the risk for internalizing problems [5, 6]. This is worrisome, as these problems are already highly prevalent among youth. Anxiety and depression, both internalizing disorders, are globally among the leading causes of mental illness and disability in children and youth [7, 8]. There are, however, also studies reporting no differences in internalizing problems or even less internalizing problems in migrant children compared to children without a migrant background [5, 9]. These inconsistencies may be explained by study differences in age groups, informants and measures. First, studies differ regarding the age range of the sample and a substantial part of the studies on differences between migrant and non-migrant children in internalizing problems include samples that cover a broad range of ages, including both children and adolescents [9–11]. Prevalence of anxiety and depression may differ across age [12], with increasing prevalence during adolescence [13, 14]. In addition, during adolescence, gender differences become more pronounced for anxiety and become visible for depression [13, 14]. Therefore it is difficult to compare the prevalence of anxiety and depression symptoms across studies covering different age ranges.

Secondly, studies differ regarding informants. Low agreement between child-reported and parent- or teacher-reported anxiety and depression has been shown [15, 16]. In addition, the ethnic background of parents or teachers has been shown to influence the identification of childhood anxiety and depression symptoms [17–19]. As anxiety and depression have a strong experiential component and self-report reflects children’s own feelings, self-report is the preferred method when studying these problems. Children’s self-reports are considered to be reliable from age 8 years onwards [20].

Thirdly, questionnaires used in previous studies are not always fit for assessing specific emotional problems. For example, the Child Behavior Checklist [CBCL; 21], the Youth Self Report [YSR; 22], and the Strengths and Difficulties Questionnaire [SDQ; 23] are measures to assess emotional problems in general. The Revised Child Anxiety and Depression Scale [RCADS; 24] was developed to measure symptoms of anxiety and depression exclusively. A disorder-specific questionnaire is able to differentiate between different types of anxiety. This is important, as the study of Austin and Chorpita [25], in which the RCADS was used, showed ethnic differences between various anxiety disorders. Therefore, studies using non-disorder-specific questionnaires may not find differences on a general anxiety/mood scale, while there may have been differences between migrant and non-migrant children for specific anxiety disorders.

Apart from these methodological factors, apparent differences in anxiety and depression scores in migrant and non-migrant children may reflect differential risk exposure. Differential exposure to discrimination and peer rejection may be among the factors underlying any apparent differences. European migrant youth and young adults report feeling discriminated [1], and compared to non-migrant children, migrant children are more often rejected by their native peers [26]. Peer rejection is linked to the development of internalizing problems [27, 28]. Further, migrant youth report more peer and social problems, and are more often involved in bullying, whether as victim or bully or both [29–31].

Another factor that may explain differences in internalizing problems between migrants and non-migrants, is family socioeconomic position (SEP). An important indicator of SEP is financial affluency. Migrant families, specifically from non-western descent, are more often less affluent than families without a migration background [32], and poverty is a risk factor for anxiety and depression in children [33, 34]. In addition, a low SEP is also a risk factor for peer rejection [35].

Lastly, no ‘one size fits all’ approach applies to migrant groups. For instance, their (general) migration history may vary, as may their cultural/language background, level of education and SEP. In the Netherlands, the three largest migrant groups are from Surinam/Netherlands Antilles (South America/Caribbean), Turkey (Middle East), and Morocco (North Africa) [36]. Surinam and the Netherlands Antilles are former colonies of the Netherlands. As schools in Surinam and the Antilles followed the Dutch curriculum, children were not only taught Dutch as first language, but also Dutch history and geography lessons. Turks and Moroccans came to the Netherlands as laborers in the 1960’s and 1970’s, mostly being men from rural areas and often with low education level. They were originally expected to work temporarily in the Netherlands and then return to their country of origin; they were called ‘guest-laborers’. As a result, little effort was made offering services that would help their societal integration, such as language courses. In practice, many of these laborers stayed in the Netherlands and had their families come over or started a family in the Netherlands. Most Dutch migrant children are the second generation, and thus born in the Netherlands. However, on population level, remnants of these differences between groups still exist. For example, in Amsterdam, the Netherlands, Surinamese/Antillean children more often have lower educated parents than Dutch children, and Turkish and Moroccan children more often have lower educated parents than Surinamese/Antillean children [37]. Also in Amsterdam, Surinamese/Antillean households are often more affluent than Turkish and Moroccan households, but less affluent than Dutch households [32]. These differences in SEP may constitute different levels of risk for child internalizing problems. As for peer rejection, in a small sample of young Dutch children, it was found that children with a Dutch background preferred Middle Eastern/North African children less than Black (in the Netherlands mostly Surinamese/Antillean) children [26].

In the present paper, we examine differences in symptoms of anxiety and depression between Dutch migrant and non-migrant children, in school-aged children from Dutch, Surinamese/Antillean, Turkish, and Moroccan background using a disorder specific, anxiety and depression self-report questionnaire, while also taking into account peer acceptance and family SEP. We hypothesized that migrant children would report more symptoms of anxiety and depression than children with a non-migration background, and that Turkish and Moroccan children would report more symptoms than Surinamese/Antillean children. Further, we expected that these differences would decrease after adjusting for peer acceptance or family SEP. Lastly, we expected to find gender differences, as many studies reported gender differences for internalizing problems among youth [5]. We examined whether this expected difference was the same across the various groups.

## Methods

### Sample

We used baseline data from a controlled trial evaluating a preventive intervention aimed at reducing symptoms of anxiety and depression [63]. Participants were primary school children from grades 4 to 6 (generally 9–12 years old) in the Amsterdam area, the Netherlands. Data were collected in school years 2010–2011 and 2011–2012.

### Measures

*Revised Child Anxiety and Depression Questionnaire (RCADS).* The RCADS is a 47-item questionnaire that measures symptoms of anxiety and depression in children [24]. The RCADS consists of six scales, based on the DSM-IV classification of childhood anxiety and depression (American Psychiatric Association, 1994): generalized anxiety disorder (GAD), social phobia (SP), separation anxiety disorder (SAD), panic disorder (PD), obsessive compulsive disorder (OCD), and major depressive disorder (MDD). The five anxiety scales can be combined into a total anxiety scale, all six scales can be combined into a total internalizing scale. Examples of items are: “I worry about things”, “I feel sad or empty”, and “I feel scared when I have to take a test”. Children indicate how often each item applies to them on a 4-point Likert scale (never, sometimes, often, always). Confirmatory factor analysis with six factors showed an acceptable fit (RMSEA 0.048, TLI 0.86) and good internal consistency (alphas ranged from 0.75 to 0.95 for the total sample and from 0.70 to 0.96 for the different migrant and non-migrant subgroups) in sample including the sample used in the present study [64]. Results from discrete multiple-group confirmatory factor analyses showed that in the present sample the RCADS scores could be meaningfully compared between boys and girls. The scores of GAD, OCD, and MDD scales could also be meaningfully compared between Dutch and Surinamese/Antillean, Turkish, and Moroccan children. For the SP (all three migrant groups compared to Dutch children), SAD (only for Turkish children compared to Dutch), and PD (only for Moroccan children compared to Dutch) scales, some minor violations of measurement invariance were found, which impacted the mean factor scores minimally [62].

*Sociodemographic information.* Children were asked to fill in their birth date and their own and parents’ country of birth, and the four digits of their postal code.

Migration status was based on the mother’s country of birth, or, if the mother was born in the Netherlands, the father’s country of birth [cf. 38]. Classification was based on the most common migrant groups in the Netherlands: Dutch, Turkish, Moroccan, Surinamese and Antillean. Type of immigration was classified as first (child was born outside the Netherlands) or second generation (child was born in the Netherlands).

A SEP score could be retrieved from the family’s 4-digit postal code. This score was based on mean household income, percentage of low household income, percentage of unemployment, percentage of households with a low education level on average. Negative numbers indicate a lower SEP and positive numbers indicate a higher SEP [39]. The composite score on the 4-digit postal code level validly reflects SEP in Dutch neighborhoods [40, 41].

*Social preference scores.* We used classroom social preference scores as a measure of peer rejection [cf. 28]. Children were asked to unlimitedly nominate classmates they liked (rejection) and did not like (popularity). Both scores were summed and divided by the number of classmates minus one, as self-nomination was not allowed. By subtracting the rejection score from the popularity score a social preference score was computed. Low social preference scores indicate poor acceptance by classmates and have been widely accepted as a valid measure of peer status [42].

### Procedures

All 265 primary schools in the Amsterdam area were invited for participation in an intervention study evaluating the effectiveness of a school-based, indicated prevention program targeting anxiety and depression [63]. The 45 participating schools were not different from the remaining schools regarding migrant background and SEP composition. Children and parents received an information letter about the study and a passive informed consent form. If children or parents did not wish to take part in the trial, they could decline to participate.

Children completed questionnaires in the classroom during school time. Researchers or research assistants explained the questionnaires and were available for additional clarification during completion of the questionnaires.

Ethical permission for the study was granted by the Medical Ethics Committee of the VU University Medical Center Amsterdam, the Netherlands.

### Analyses

Descriptive statistics were performed using SPSS Statistics version 19 (IBM SPSS Statistics, 2010). Differences between children with and without information on SEP were assessed using chi-square tests and a t-test.

Differences in SEP, social preference scores, anxiety, and depression were assessed using linear multilevel regression analyses, as children were nested within classes and classes within schools (Stata version 15.1 Intercooled; StataCorp LLC). The first series of models assessed associations between RCADS scales and migrant group, adjusting for gender and age. The next series of models were additionally adjusted for SEP or social preference score. Gender differences were derived from the first model. Moderating effects of gender were investigated by adding a migrant x gender interaction term to Model 4. We used a cut-off value of *p* < 0.10 for testing the significance of moderating effects [cf. 43].

## Results

### Sample

Of the 3890 invited children from grades 4, 5 and 6, 115 (3%) declined. Another 139 children (4%) dropped out before or during data collection because of leaving school, illness or unknown reasons. Children with another background than Dutch, Surinamese/Dutch Antillean, Turkish, or Moroccan (n = 826; 24%) or missing background (*n* = 124; 3%) were excluded. Children for whom no information on gender (*n* = 3; 0.1%) and/or SEP (*n* = 621; 23%) was available were excluded from the present analyses. Children without information on SEP were significantly more often boys (28% boys versus 18% girls with missing SEP; χ^2^ < 0.01) and were younger (mean age: 10.3 versus 10.7 years; *p* < 0.01). Missing information on SEP was not selective for background (χ^2^ = 0.45).

The distribution in the original sample – which also included children from other descent than the four largest migration groups – was 40% Dutch, 12% Surinamese/Antillean, 9% Turkish, 16% Moroccan, and 24% other background (total 101% due to rounding) [64]. The distribution of background in the present sample was 53% Dutch, 15% Surinamese/Dutch Antillean, 11% Turkish, and 21% Moroccan. The majority (94%) of the children were born in the Netherlands. Table 1 reports the characteristics of our sample per group.

**Table 1:** Demographic characteristics, means (standard deviations) and item means of the RCADS scales in the study sample of children aged 8–13 years, per migration group

	Dutch *n* = 1086 *M (SD)*		Surinamese/ Antillean *n* = 310 *M (SD)*		Turkish *n* = 235 *M (SD)*		Moroccan *n* = 432 *M (SD)*	
Age	10.7 (0.9)		10.9 (0.8)		10.8 (0.8)		10.6 (0.8)	
Gender *n* girls (%)	608 (56%)		182 (59%)		116 (49%)		235 (54%)	
Generation *n* 2nd (%)	NA		263 (85%)		213 (91%)		375 (87%)	
SEP^a^	0.41 (1.26)		-1.65 (1.25)		-1.71 (1.35)		-1.66 (1.18)	
Social preference^b^	0.15 (0.25)		0.21 (0.27)		0.13 (0.25)		0.10 (0.27)	
Scale (range) Item means	Boys	Girls^c^	Boys	Girls^c^	Boys	Girls^c^	Boys	Girls^c^
Total anxiety (0-111)	17.0 (13.0)	23.8 (16.4)	20.2 (15.3)	26.3 (17.6)	22.6 (14.5)	26.3 (17.1)	21.1 (18.1)	28.7 (21.8)
	0.46	0.64	0.54	0.71	0.61	0.71	0.60	0.77
GAD (0–18)	3.4 (2.9)	4.4 (3.3)	4.0 (3.6)	4.8 (3.7)	4.1 (3.1)	5.0 (3.9)	4.3 (4.0)	5.3 (4.5)
	0.57	0.74	0.67	0.80	0.69	0.84	0.72	0.88
SP (0–27)	5.9 (4.1)	8.5 (5.1)	6.4 (4.8)	8.6 (5.7)	7.1 (4.7)	8.9 (5.3)	6.6 (5.6)	9.0 (6.0)
	0.66	0.95	0.71	0.95	0.79	0.99	0.74	0.99
SAD (0–21)	1.8 (2.5)	3.3 (3.3)	1.7 (2.5)	2.8 (3.2)	2.2 (2.4)	3.3 (3.5)	2.4 (3.3)	3.8 (4.1)
	0.26	0.47	0.25	0.40	0.31	0.47	0.34	0.54
PD (0–27)	3.0 (3.2)	4.3 (4.2)	3.8 (3.5)	5.3 (4.8)	4.5 (4.2)	5.0 (4.1)	4.0 (4.5)	5.8 (5.5)
	0.33	0.48	0.43	0.59	0.51	0.56	0.45	0.64
OCD (0–18)	2.9 (2.7)	3.3 (3.2)	4.2 (3.6)	4.8 (3.6)	4.6 (3.5)	4.2 (3.4)	4.0 (3.7)	4.6 (4.1)
	0.48	0.55	0.70	0.80	0.77	0.70	0.67	0.77
MDD (0–30)	5.1 (3.6)	5.8 (4.0)	4.8 (3.6)	5.9 (4.1)	5.9 (4.1)	5.7 (4.3)	4.8 (4.3)	5.6 (4.3)
	0.51	0.58	0.48	0.59	0.57	0.56	0.48	0.56

*Note:* SEP = socioeconomic position. RCADS = Revised Child Anxiety and Depression Scale. GAD = Generalized Anxiety Disorder. SP = Social Phobia. SAD = Separation Anxiety Disorder. PD = Panic Disorder. OCD = Obsessive Compulsive Disorder. MDD = Major Depressive Disorder. *n* varies slightly per scale due to missing values. ^a^ =all ethnic minority groups had lower SEP scores (*p* < 0.05). ^b^ = Turkish and Moroccan children had lower social preference scores than Dutch and Surinamese children (*p* < 0.05). ^c^ = All girls had significantly higher scores than boys (*p* < 0.05)

### Differences Between Migrant and Non-Migrant Groups

Table 1 presents the raw means and standard deviations of the RCADS scales per group. Item means were added to enhance comparison of scale scores. All migrant groups had significantly lower family SEP scores than the Dutch group. Turkish and Moroccan children had significantly lower social preference scores than Dutch and Surinamese/Antillean children.

Multilevel analyses adjusted for gender and age showed that migrant children reported significantly higher anxiety scores, specifically PD and OCD, than Dutch children without a migrant background (Model 1, Table 2). Moroccan children also reported higher total anxiety and GAD scores. When further adjusting for SEP, all migrant groups reported higher OCD scores than Dutch children (Model 2). In addition, Moroccan children reported more GAD and PD scores compared to Dutch children, and Surinamese children reported higher PD scores than Dutch children. Adjusting for social preference scores influenced the differences in anxiety scores less than adjusting for SEP (Model 3). In these analyses, all migrant groups reported more OCD symptoms than Dutch children. Surinamese/Antillean children also reported higher PD scores compared to Dutch children. Moroccan children also reported higher total anxiety, GAD, and PD scores, and lower depression scores compared to Dutch children. The final model (Model 4), adjusting for age, gender, SEP, and social preference, showed that higher OCD scores were reported in all ethnic minority groups than in Dutch children, and that higher PD scores were more often reported in Surinamese/Antillean and Moroccan children than in Dutch children. Moroccan children reported lower depression scores than Dutch children. As a sensitivity analysis to examine the influence of first generation migrant children, we reran the model without these children (Model 5). Although the general picture is that anxiety scores were lower without this group, only PD symptoms in Surinamese/Antillean children were no longer significantly higher compared to Dutch children, and depression scores in Moroccan children became significantly lower than in Turkish children.

**Table 2:** Differences in RCADS scores in Dutch children aged 8–13 years from different migration groups: Results of multilevel linear regression analyses

Scale	Dutch	Surinamese/ Antillean *B* [95% CI]	Turkish *B* [95% CI]	Moroccan *B* [95% CI]
Model 1: Adjusted for age and gender				
Total anxiety (0-111)	ref	1.87 [-0.66, 4.39]	2.34 [-0.45, 5.13]	2.81 [0.49, 5.13] ^a^
GAD (0–18)	ref	0.42 [ -0.10, 0.93]	0.50 [-0.06, 1.07]	0.66 [0.20, 1.12] ^a^
SP (0–27)	ref	-0.01 [-0.78, 0.76]	0.45 [-0.40, 1.30]	0.22 [-0.48, 0.92]
SAD (0–21)	ref	-0.30 [-0.78, 0.17]	-0.10 [-0.62, 0.43]	0.22 [-0.20, 0.66]
PD (0–27)	ref	0.82 [0.19, 1.45] ^a^	0.74 [0.04, 1.44] ^a^	0.97 [0.40, 1.54] ^a^
OCD (0–18)	ref	1.17 [0.67, 1.67] ^a^	1.02 [0.47, 1.57] ^a^	0.98 [0.52, 1.43] ^a^
Depression (0–30)	ref	-0.01 [-0.67, 0.46]	0.15 [-0.47, 0.77]	-0.39 [-0.89, 0.12]
Model 2: Adjusted for age, gender, and SEP				
Total anxiety (0-111)	ref	1.37 [-1.28, 4.02]	1.84 [-1.08, 4,76]	2.29 [-0.19, 4.76]
GAD (0–18)	ref	0.29 [-0.25, 0.84]	0.37 [-0.23, 0.97]	0.53 [0.02, 1.04] ^a^
SP (0–27)	ref	-0.11 [-0.92, 0.71]	0.35 [-0.55, 1.25]	0.12 [-0.64, 0.88]
SAD (0–21)	ref	-0.37 [-0.88, 0.13]	-0.17 [-0.72, 0.38]	0.15 [-0.31, 0.62]
PD (0–27)	ref	0.71 [0.04, 1.38] ^a^	0.63 [-0.10, 1.37]	0.86 [0.24, 1.48] ^a^
OCD (0–18)	ref	0.99 [0.46, 1.51] ^a^	0.83 [0.25, 1.41] ^a^	0.78 [0.30, 1.27] ^a^
Depression (0–30)	ref	-0.27 [-0.89, 0.34]	-0.02 [-0.69, 0.65]	-0.56 [-1.12, 0.00]
Model 3: Adjusted for age, gender, and social preference				
Total anxiety (0-111)	ref	1.94 [-0.58, 4.46]	2.12 [-0.67, 4.92]	2.44 [0.12, 4.77] ^a^
GAD (0–18)	ref	0.42 [-0.09, 0.94]	0.45 [-0.12, 1.02]	0.59 [0.12, 1.05] ^a^
SP (0–27)	ref	0.02 [-0.75, 0.79]	0.39 [-0.46, 1.25]	0.12 [-0.58, 0.83]
SAD (0–21)	ref	-0.29 [-0.76, 0.19]	-0.10 [-0.63, 0.43]	0.20 [-0.24, 0.63]
PD (0–27)	ref	0.83 [0.20, 1.46] ^a^	0.69 [-0.01, 1.39]	0.87 [0.30, 1.45] ^a^
OCD (0–18)	ref	1.18 [0.68, 1.68] ^a^	0.97 [0.42, 1.52] ^a^	0.90 [0.45, 1.36] ^a^
Depression (0–30)	ref	-0.05 [-0.61, 0.51]	0.09 [-0.53, 0.70]	-0.53 [-1.03, -0.03] ^a^
Model 4: Adjusted for age, gender, SEP, and social preference				
Total anxiety (0-111)	ref	1.41 [-1.24, 4.06]	1.59 [-1.33, 4.51]	1.87 [-0.62, 4.36]
GAD (0–18)	ref	0.29 [-0.26, 0.84]	0.31 [-0.29, 0.91]	0.44 [-0.06, 0.95]
SP (0–27)	ref	-0.09 [-0.91, 0.73]	0.28 [-0.62, 1.18]	-0.09 [-0.91, 0.73]
SAD (0–21)	ref	-0.36 [-0.87, 0.14]	-0.18 [-0.73, 0.37]	0.12 [-0.35, 0.59]
PD (0–27)	ref	0.71 [0.04, 1.38] ^a^	0.58 [-0.16, 1.31]	0.75 [0.04, 1.38] ^a^
OCD (0–18)	ref	0.99 [0.46, 1.52] ^a^	0.77 [0.19, 1.35] ^a^	0.69 [0.21, 1.52] ^a^
Depression (0–30)	ref	-0.25 [-0.86, 0.36]	-0.12 [-0.78, 0.55]	-0.73 [-1.29, -0.17] ^a^
Model 5: Adjusted for age, gender, SEP, and social preference (first generation migrant children excluded)				
Total anxiety (0-111)	ref	1.03 [-1.66, 3.72]	2.14 [-0.80, 5.07]	1.69 [-0.83, 4.22]
GAD (0–18)	ref	0.15 [-0.42, 0.71]	0.36 [-0.26, 0.97]	0.42 [-0.11, 0.94]
SP (0–27)	ref	-0.21 [-1.04, 0.63]	0.53 [-0.38, 1.44]	-0.01 [-0.79, 0.76]
SAD (0–21)	ref	-0.37 [-0.89, 0.14]	-0.14 [-0.70, 0.42]	0.02 [-0.46, 0.50]
PD (0–27)	ref	0.63 [-0.06, 1.31]	0.63 [-0.11, 1.38]	0.73 [0.09, 1.37] ^a^
OCD (0–18)	ref	0.92 [0.39, 1.45] ^a^	0.90 [0.33, 1.48] ^a^	0.68 [0.19, 1.18] ^a^
Depression (0–30)	ref	-0.33 [-0.96, 0.30]	0.03 [-0.66, 0.71]	-0.77 [-1.35, -0.19] ^a, b^

*Note:* RCADS = Revised Child Anxiety and Depression Scale. GAD = Generalized Anxiety Disorder. SP = Social Phobia. SAD = Separation Anxiety Disorder. PD = Panic Disorder. OCD = Obsessive Compulsive Disorder. Ref = reference category. SEP = socioeconomic position. *n* varies slightly per scale due to missing values. ^a^ Significantly different from Dutch children (*p* < 0.05). ^b^ Significantly different from Turkish children (*p* < 0.05)

### Gender Differences

Girls reported more symptoms of anxiety and depression than boys (Table 1). We found no significant moderating effects of gender, indicating that gender differences were comparable for each group.

## Discussion

The present study examined differences in symptoms of self-reported anxiety and depression in migrant and non-migrant preadolescent children. We found that migrant children reported higher anxiety scores than Dutch children without a migrant background, in particular higher PD and OCD symptoms. Differences were small, but significant. Peer rejection and family SEP partly explained differences between migrant and non-migrant groups; but most differences in PD and OCD remained statistically significant after controlling for these factors. In contrast to our hypothesis, Surinamese/Antillean children did not report lower anxiety scores than their Turkish and Moroccan peers. Also unexpectedly, for MDD we did not find differences in scores between Surinamese/Antillean, Turkish and Dutch children. When taking peer rejection and family SEP into account, Moroccan children’s MDD scores were even lower than those of Dutch. Gender differences were as expected. Girls reported more symptoms of anxiety and depression than boys, and this finding was consistent across all groups.

Differences between migrant and non-migrant groups were mainly found on specific disorder scales. This could explain why studies using broad anxiety or internalizing instruments or scales did not pick up differences in specific areas in the internalizing spectrum, specifically in samples including preadolescents. As anxiety prevalence increases with age and anxiety symptoms at a young age are related to anxiety in later life [13], differences between groups with a different background may get more pronounced with age and only then become detectable using broad-band scales. Two anxiety types were more prevalent in children from all three migrant groups: PD and OCD. This finding is partly in line with a previous study by Austin and Chorpita using the RCADS in children and adolescents [25]. Both types of anxiety disorder seem to reflect the indirect expression of anxiety rather than presenting feelings of anxiety as such. As often discussed [e.g., 44], in non-Western cultures expression of anxiety and fears through somatic complaints as seen in PD or through ritualistic behavior as seen in OCD seem more common and acceptable forms than right-out statements of negative feelings or social inhibition. For OCD, other studies also found a higher prevalence in non-White samples [45, 46]. It may be that differences between migrant and non-migrant groups in OCD are a reflection of anxiety related to religious rituals and beliefs, to which OCD symptoms are linked [47]. The migrant groups included in our study are reported to be more religious than the Dutch without a migration background [48–50]. The appearance of OCD symptoms seems not related to a specific religion [47]. That could hold in the present study: where Turks and Moroccans are mainly Muslim, Surinamese are mostly Christian, Hindu or Muslim, and Antilleans mainly Christian [48].

Remarkable was the absence of differences in depression scores for Surinamese/Antillean and Turkish children compared to Dutch children, and even more surprising were the lower depression scores in Moroccan children compared to Dutch children. Given the high comorbidity between anxiety and depression [51], one would expect to find higher levels of depression symptoms in groups with more anxiety symptoms. It is unlikely that this finding is related to methodological factors of the questionnaire, as no evidence was found for migrant group-related differential item functioning of the MDD scale [62]. It could be that (expectable) migration related differences in depression scores only become visible in adolescence, given that the prevalence of depression rises during this age period [14]. In Dutch studies including older adolescents and adults, Turkish immigrants reported more depressive disorder than both those from Dutch and Moroccan groups [52–54].

Our two potential explanatory variables for ethnic differences partly explained the observed differences between groups in anxiety scores. As expected, we found that family SEP was significantly lower in migrant groups, while social preference was lower among Turkish and Moroccan children compared to Dutch children. Although a lower SEP is a risk factor for mental health problems and contributes to differences in mental health [5], like ours, other studies also found that SEP cannot fully explain mental health differences between migrant and non-migrant groups [55]. However, there are many definitions and measures of SEP. Peer rejection hardly explained differences in anxiety and depression scores. Previous research linked peer rejection to internalizing problems [27, 28]. However, as these studies investigated this relation over a longer time period, it may be difficult to find the same link in a cross-sectional study like ours.

### Study Implications

The findings of this study suggest that children from migrant families are at elevated risk for some types of anxiety problems, and qualify for mental health care at least as much as children from non-migrant families. Despite this, these problems often seem to go unnoticed to both parents and mental health professionals. Several studies have shown that for young children and adolescents in the Netherlands, a migration background is associated with less mental health care use [19, 56]. This may be partly due to the fact that migrant parents report less internalizing problems than Dutch parents [57, 58]. A lack of internalizing problem identification by parents was found to be an important mediator between migrant status and mental health care use [19]. Moreover, there is evidence that child health care professionals have a lower identification rate of psychosocial problems in children with a migrant background. For example, professionals identified only 30% of Turkish and Moroccan children with parent-reported elevated psychosocial problem scores, compared to 60% of Dutch children or children from comparable countries to the Netherlands [59]. Our study findings underscore the importance of using child-reported data on internalizing problems, in research [60], as well as in practice, as they might go unnoticed if only parents’ or clinicians’ views are used.

Problem identification should be followed by effective and accessible mental health care. In the Netherlands, a migration background is associated with less mental health care use in young children and adolescents [19, 56]. It seems that differences in beliefs about emotional problems and attitudes toward mental health care play a role in this, as it was found that Turkish and Moroccan parents advocated less active solutions towards their children’s internalizing problems, and that Surinamese and Moroccan parents had more fear towards mental health care compared to Dutch parents [61]. This fear appeared to be mainly driven by the expected shame to the family if mental health problems would be found out by others. School-based mental health interventions may overcome these problems, by offering equal access for all students in a non-stigmatizing environment. Indeed, a Dutch study into a school-based preventive intervention for children with elevated anxiety or depression scores included children from various migrant groups, and showed that the program was equally effective among Dutch children with or without a migrant background compared to a non-intervention control group, up to twelve months post-intervention [63]. Further, migrant children appraised the program equally enjoyable, and even more useful than Dutch children [65].

In addition, our findings suggest the need for further research into potential differences in the nature, background and forms of expression of anxiety problems in children from different migrant groups, given the notable differences in symptoms of PD and OCD, but not other types of anxiety.

Another note for further research regards the study of the immediate impact of migration on children from migrant families. Our sample consisted largely of second generation migrant children (i.e., born in the Netherlands, but with one or two foreign-born parents). We performed a sensitivity analysis to examine whether outcomes were different if first generation migrant children were excluded. The pattern of higher anxiety scores in migrant children remained largely the same. It is nevertheless important to define migrant generation status – as this is unclear in many previous studies [60] – and to examine immediate effects of migration on types of anxiety problems and potential differences in these between children from different migration generations, which have been found before in adolescent migrant samples [6].

### Strengths and Limitations

To our knowledge, this is the first study examining differences in anxiety and depression scores in a large sample of pre-adolescent migrant and non-migrant children, using an anxiety and depression disorder specific self-report questionnaire, which also took into account family SEP and peer rejection using standardized measures. The large sample of school-aged children allowed us to differentiate between the four largest migrant and non-migrant groups in the Netherlands, rather than comparing children with and without a migration background only. This is particularly important, as our study showed that there are differences in demographic characteristics as well as in symptoms per group.

The low non-response rate (7%) can be regarded as an important strength of our study. However, the sample in the present study was smaller than the original sample [64], because children for whom no information on gender, migration background and/or SEP was available (*n* = 747) were excluded from the analyses. Children for whom no information about family SEP was available were younger and more often boys. However, as missing family SEP was not related to migration background, these missing values are unlikely to have influenced our findings.

Regarding measures our study has three limitations. As our study design did not allow us to collect data from parents directly, our measure of family SEP was calculated per postal code area and not based on parent reports of individual household income or parental educational level. Children’s report of the postal code may have been more prone to error than when collected from the parents. Secondly, our definition of migration background did not allow us to take a bicultural background into account and was only limited for first en second generation migrant status. Finally, for some subscales (SP, SAD, and PD) scales, in this sample some minor violations of measurement invariance for some migrant groups were found [62]. However, these impacted the mean factor scores minimally.

## Conclusion

Dutch school-aged children from migrant groups self-reported higher scores for specific anxiety problems than their Dutch peers without migrant background, while depression scores were similar or lower. Differences between groups were partly explained by family SEP and peer rejection. Differences were small but significant. Nonetheless, as the prevalence of anxiety increases with age [13], small differences in school-aged children may become larger in later life, and differences in depression scores may occur later in adolescence. Disorder specific and self-report questionnaires can provide more detailed information about differences in specific anxiety types and help to identify those children in need of mental health services. We recommend more research into the causes of differences between migrant and non-migrant children as well as accessible prevention programs to prevent an increase in anxiety and depression.

## Summary

Migrant children form a substantial part of today’s society. The majority of literature shows that internalizing problems, among which anxiety and depression, are more prevalent in migrant children. Some studies, however, did not find differences between children with and without a migration background. Methodological factors may play a role in these inconsistencies. Therefore, we included a specific age group (i.e., pre-adolescent children), and used the Revised Child Anxiety and Depression Scale (RCADS), a self-report questionnaire that measures symptoms of five DSM-IV-classified anxiety disorders and depression. Our large sample (*n* = 2063) consisted of children from the four largest migrant and non-migrant groups in the Netherlands: Dutch, Surinamese/Antillean, Turkish, and Moroccan. Most migrant children were second generation migrants, i.e., they were born in the Netherlands but their parents were not. Peer rejection and socioeconomic position, which are often less favorable in migrant children, were taken into account as potential explanatory factors. We found that Surinamese/Antillean, Turkish, and Moroccan children reported significantly higher anxiety scores than Dutch children. SEP and peer rejection partly explained higher anxiety scores. Surinamese/Antillean and Turkish children reported comparable depression scores to Dutch children, but Moroccan children reported lower depression scores after adjusting for SEP and peer rejection. Girls reported higher anxiety and depression scores across groups. Differences between children with and without a migration background were small, but these may become larger in later life as the prevalence of anxiety disorder increases with age. It may be that the sample was too young to detect more differences in depression scores, as the prevalence rises in adolescence, although it may also be possible that different patterns in depression prevalence exist. We recommend using self-reported and disorder specific data in research and practice as well as additional research into the causes of differences between migrant and non-migrant children. Further, the outcomes of the present study underscore the importance of equal access to inclusive prevention programs focusing on anxiety and depression across groups.
