# Correction to: Differences in Anxiety and Depression Among Migrant and Non-Migrant Primary School Children in The Netherlands

**DOI:** 10.1007/s10578-022-01472-y

**Published:** 2022-11-28

**Authors:** Mia P Kösters, Mai JM Chinapaw, Marieke Zwaanswijk, Marcel F van der Wal, Hans M. Koot

**Affiliations:** 1 Healthy Living Department, Public Health Service of Amsterdam, Amsterdam, The Netherlands; 2 Amsterdam UMC location Vrije Universiteit Amsterdam, dept Public and Occupational Health, Amsterdam, The Netherlands; 3 Amsterdam Public Health Research Institute, Amsterdam, The Netherlands; 4 Dutch Knowledge Centre for Child and Adolescent Psychiatry, Utrecht, The Netherlands; 5 Department of Clinical, Neuro- and Developmental Psychology, Amsterdam Public Health Research Institute, Amsterdam, The Netherlands; 6 Department of Clinical, Neuro- and Developmental Psychology, Vrije Universiteit Amsterdam, Van der Boechorststraat 7, 1081 BT Amsterdam, The Netherlands

**Correction to: Child Psychiatry & Human Development**

10.1007/s10578-022-01454-0

In the original versions of this article, published online on 02 November 2022, the references to the authors’ own work were inadvertently published with “[blinded for peer review]”. These references have been added in the corrected versions. The original article has been corrected. 

